# Antineoplastic Effect of ALK Inhibitor Crizotinib in Primary Human Anaplastic Thyroid Cancer Cells with STRN–ALK Fusion In Vitro

**DOI:** 10.3390/ijms25126734

**Published:** 2024-06-19

**Authors:** Silvia Martina Ferrari, Francesca Ragusa, Giusy Elia, Valeria Mazzi, Eugenia Balestri, Chiara Botrini, Licia Rugani, Armando Patrizio, Simona Piaggi, Concettina La Motta, Salvatore Ulisse, Camilla Virili, Alessandro Antonelli, Poupak Fallahi

**Affiliations:** 1 Department of Clinical and Experimental Medicine, University of Pisa, 56126 Pisa, Italy; 2 Department of Surgery, Medical and Molecular Pathology and Critical Area, University of Pisa, 56126 Pisa, Italy; francesca.ragusa@phd.unipi.it (F.R.); giusy.elia@phd.unipi.it (G.E.); mazzivaleria@gmail.com (V.M.); eugenia.balestri@phd.unipi.it (E.B.); chiara.botrini@gmail.com (C.B.); licia.rugani99@gmail.com (L.R.); alessandro.antonelli@unipi.it (A.A.); 3 Department of Emergency Medicine, Azienda Ospedaliero-Universitaria Pisana, 56126 Pisa, Italy; armandopatrizio125@gmail.com; 4 Department of Translational Research and New Technologies in Medicine and Surgery, University of Pisa, 56126 Pisa, Italy; simona.piaggi@unipi.it (S.P.); poupak.fallahi@unipi.it (P.F.); 5 Department of Pharmacy, University of Pisa, 56126 Pisa, Italy; concettina.lamotta@unipi.it; 6 Department of Surgery, “Sapienza” University of Rome, 00161 Rome, Italy; salvatore.ulisse@uniroma1.it; 7 Department of Medico-Surgical Sciences and Biotechnologies, Endocrinology Section, “Sapienza” University of Rome, 04100 Latina, Italy; camilla.virili@uniroma1.it

**Keywords:** anaplastic thyroid cancer, crizotinib, primary cell cultures, tyrosine kinase inhibitors, proliferation, apoptosis, migration, invasion, STRN–ALK fusion

## Abstract

Anaplastic thyroid cancer (ATC) is one of the deadliest human cancers and represents <2% of thyroid carcinomas. A therapeutic target for ATC is represented by anaplastic lymphoma kinase (ALK) rearrangements, involved in tumor growth. Crizotinib is an oral small-molecule tyrosine kinase inhibitor of the ALK, MET, and ROS1 kinases, approved in ALK-positive non-small cell lung cancer. Until now, the effect of crizotinib in “primary human ATC cells” (pATCs) with transforming striatin (STRN)–ALK fusion has not been reported in the literature. In this study, we aimed to obtain pATCs with STRN–ALK in vitro and evaluate the in vitro antineoplastic action of crizotinib. Thyroid surgical samples were obtained from 12 ATC patients and 6 controls (who had undergone parathyroidectomy). A total of 10/12 pATC cultures were obtained, 2 of which with transforming STRN–ALK fusion (17%). Crizotinib inhibited proliferation, migration, and invasion and increased apoptosis in 3/10 pATC cultures (2 of which with/1 without STRN–ALK), particularly in those with STRN–ALK. Moreover, crizotinib significantly inhibited the proliferation of AF cells (a continuous cell line obtained from primary ATC cells). In conclusion, the antineoplastic activity of crizotinib has been shown in human pATCs (with STRN–ALK) in preclinical studies in vitro, opening the way to future clinical evaluation in these patients.

## 1. Introduction

Thyroid cancer (TC) is the most prevalent among endocrine tumors worldwide, and in recent years, its incidence has been growing [1,2,3,4,5,6].

TC classification depends on the cells of origin, and its incidence varies in relation to the histotype. Differentiated TC (DTC) derives from thyroid follicular cells and is represented by follicular TC (FTC) and papillary TC (PTC) for ~85–95% of TCs. Hürthle cells TC and poorly differentiated TC (PDTC) represent ~2–5% and anaplastic TC (ATC) ~1.7% of TCs. Medullary TC (MTC) arises from para-follicular C cells of neuroendocrine origin and represents ~3–5% of TCs [7,8,9,10,11].

The first kind of treatment for patients with DTC and MTC is surgery. After thyroidectomy, in DTC, radioactive iodine (RAI) is administered to ablate the residual normal thyroid, and it also permits to treat subclinical micrometastatic disease and/or residual or metastatic TC [12]. At follow-up, neck ultrasound and measurement of basal and thyroid-stimulating hormone (TSH)-stimulated thyroglobulin (Tg) are performed every 3–6 months in the 1st year and then at different intervals according to the initial risk assessment [13].

The prognosis of PDTC and ATC is worse than that of DTC [14]. PDTC and ATC have also lower overall survival (OS) rates, with mean survival of ~3.2 years and 6 months, respectively [15].

Distant metastases (in bone, lung, and other locations) are present in ~5% of DTC patients at diagnosis, while during follow-up, ~15% of patients develop recurrences, and the median survival is reduced from 70% to 50% after 10 years [16,17]. When DTC progresses, the iodide uptake by thyrocytes is abolished, and cells do not respond to RAI, with negative effects on the prognosis [18]. For patients with metastatic DTC, therapeutic options (such as surgery, chemotherapy, and external beam radiation therapy), have a palliative role, without prolongation of survival when used either alone or combined [19].

For these reasons, it is a hurdle task to make a prediction of responsiveness to clinical therapy in ATC patients, and it could be useful to determine an effective systemic treatment in these patients to ameliorate their quality of life [20,21].

The new knowledge of the molecular pathways involved in the development of ATC has given rise to the realization of novel drugs.

Whole-genome [22], whole-exome [23], and targeted sequencing studies [15,24] have been conducted to understand the genetic changes in ATC.

The multistep oncogenesis model is the commonly accepted model of TC oncogenesis, according to which mature follicular cells can turn into DTC cells and then progress into undifferentiated TC cells [25,26]; however, the molecular mechanisms that guide progression to a more aggressive pattern are not completely explained [22].

In TC, ~90% of alterations are the reciprocally exclusive activating oncogenes RAS (~13%) and BRAF (~60%) and rearrangements [RET, anaplastic lymphoma kinase (ALK), and NTRK genes], whilst the other 10% are loss-of-function mutations of tumor suppressor genes (i.e., TP53, PTEN, and PPARγ) [27,28,29].

Mutations including the TERT promoter, TP53, eukaryotic translation initiation factor 1A X-linked (EIF1AX), genes taking part in the PIK3CA-AKT-mTOR pathway, SW1/SNF complex, and mismatch repair genes are linked to higher aggressiveness in TC [30,31,32]. The passage from follicular thyrocytes to PTC or FTC is guided by mutations that activate the MAPK or PI3K-AKT signaling pathways, and progression to PDTC is linked to further mutations and rearrangements. ATC is also characterized by epigenetic alterations and infiltration of immune cells, which create an intracellular microclimate that favors genetic instability and oncogenesis [25,33].

The PI3K-AKT and MAPK pathways can lead to the activation of the Wnt/β-catenin pathway, which regulates growth and stem cell differentiation, and they can also induce FOXO- and NF-κB pathways. Furthermore, the HIF1α-pathway takes part in angiogenesis and modified cell metabolism [34,35]. Catenin Beta 1 (CTNNB1) is involved in cell adhesion and invasiveness, and HIF1 strongly activates vascular endothelial growth factor A (VEGFA).

The role of ALK rearrangements in ATC is still unclear. ALK rearrangements caused by mutations and fusions can activate both the RAS-BRAF-MEK pathway and the PI3K-AKT-mTOR pathway in cancers, such as in non-small cell lung carcinoma (NSCLC) [36,37,38]. ALK fusions, predominantly with striatin (STRN) or echinoderm microtubule-associated protein-like 4 (EML4), are reported in 1–3% of PTCs and much frequently in PDTC [39,40,41,42,43]. STRN–ALK fusion takes part in progression from PTC to PDTC [44] and have been detected in up to 20% of ATCs [15,22,45].

ALK fusions are considered both a diagnostic marker and a possible therapeutic target, as shown by different case reports of patients with ALK-positive TC, including cases of metastatic and RAI-resistant cancer treated with crizotinib [45,46,47].

Small-molecule inhibitors of tyrosine kinases (TKIs), which are involved in ATC progression, have been conceived recently, and some of them have been approved in the US (FDA) and EU (EMA) to treat aggressive and refractory TCs [48]. Sorafenib [49], lenvatinib [50], and cabozantinib [51] were approved to treat recurrent or metastatic RAI-refractory DTC, cabozantinib [52] and vandetanib [53,54] for MTC, and more recently, the combined treatment with dabrafenib and trametinib for ATC with V600EBRAF mutation [55,56,57].

In vitro, we showed the antitumoral effect of lenvatinib [58] and vandetanib [59] in primary human ATC cell (pATC) cultures, established based on both biopsy or fine-needle aspiration (FNA) cytology [60], and in vivo, in xenotrasplants of ATC in nude mice. Moreover, we demonstrated the antineoplastic effect of pazopanib in human pATCs in vitro [48]. We also reported the antiangiogenic and antitumoral action of the pyrazolo [3,4-d]pyrimidine compounds CLM3 (with antiangiogenic activity and able to inhibit EGFR, VEGFR, and RET TK), in primary ATC cultures [61], and CLM29 and CLM24, in pATCs and in the 8305C continuous cell line [62]. The cyclic amide CLM94 had an antitumoral activity in vitro in pATCs and in vivo in CD nu/nu mice [63].

The use of in vitro drug testing could give indications to clinicians regarding which could be the right therapy to avoid the administration of ineffective compounds, since it is possible to predict clinical benefits through disease-oriented in vitro drug screening [64,65]. A positive predictive value (PV) of 60% and a negative PV of 90% have been shown in human tumoral cell lines in vitro [66].

Crizotinib is an oral small-molecule TKI of the ALK, MET, and ROS1 kinases, approved in ALK-positive NSCLC and, as written above, used to treat patients with ALK-positive TC, including cases of metastatic and RAI-resistant cancer [45,46,67].

In this study, we aimed to assess in vitro the antineoplastic effect of crizotinib in primary ATC cells with/without STRN–ALK fusion.

## 2. Results

The absence of expression of NIS, TSH receptor, TPO, and Tg was reported by immunohistochemistry, while immunocytochemistry revealed slight and focal positivity to cytokeratin. A pattern identical to initial neoplastic tissue was shown by DNA fingerprinting. Mutations in BRAF, N-RAS, H-RAS, K-RAS, RET/PTC1, RET/PTC3, and PAX8/PPARγ rearrangements were not detected, while STRN–ALK fusion was reported in 2/12 (17%) samples.

From 12 ATC patients, 10 pATC cultures were established, and the inhibition of proliferation, migration, and invasion and an increase in apoptosis were demonstrated in 3/10 pATC cultures (2 of which with/1 without STRN–ALK), particularly in those with STRN–ALK.

### 2.1. Proliferation, Cell Viability, and Cytotoxicity Assay

Inhibition of proliferation was demonstrated in 3/10 pATC cultures (2 of which with/1 without STRN–ALK), particularly in those with STRN–ALK.

**Figure 1:**
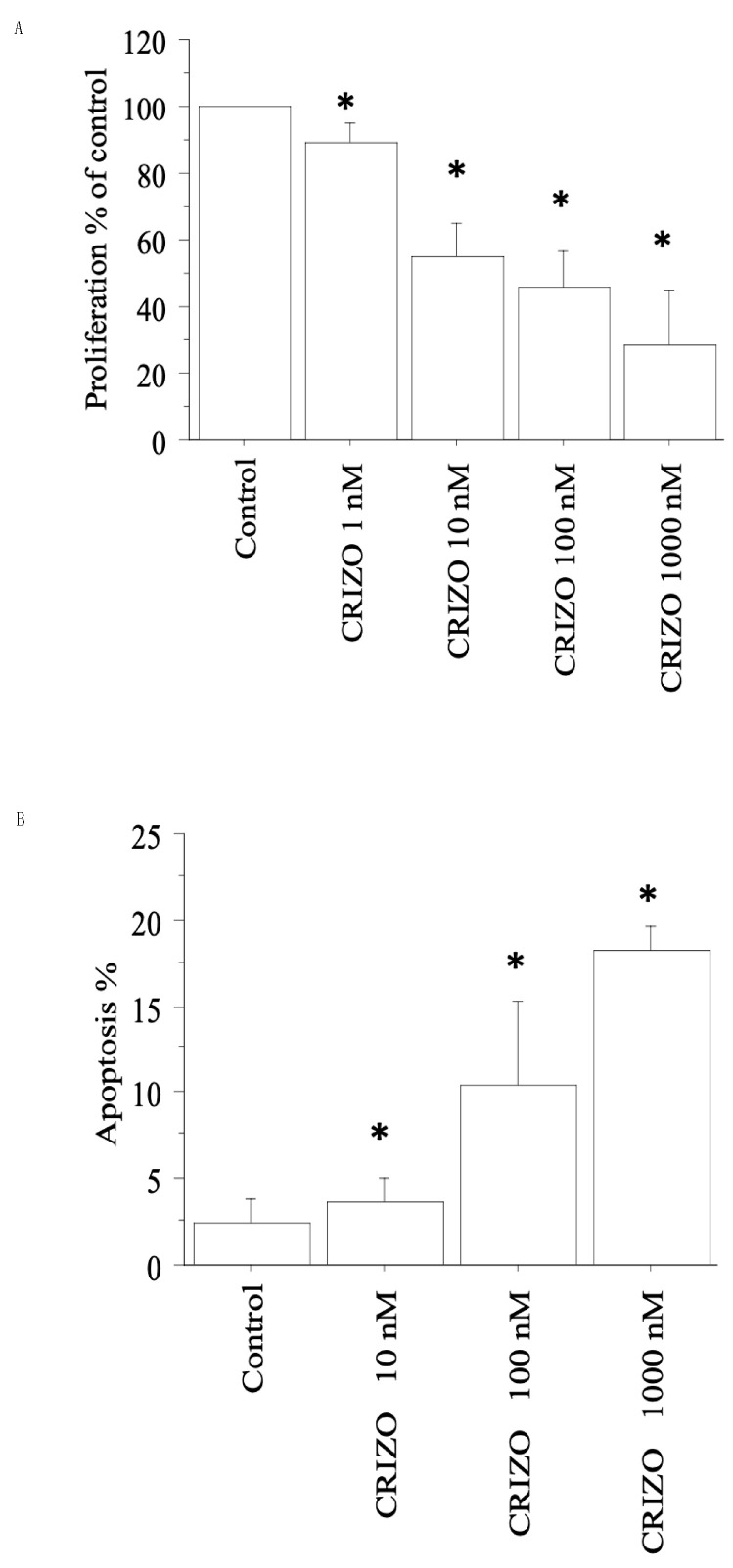
Cell proliferation and apoptosis assays in the first pATC culture with STRN–ALK fusion. (**A**) WST-1 assay in pATCs treated for 24 h with crizotinib (1, 10, 100, and 1000 nM). Proliferation was significantly reduced vs. Control. Bars represent means ± SD. * *p* < 0.05 vs. Control. (**B**) Apoptosis was shown by Hoechst 33342 staining in pATCs after treatment with crizotinib (10, 100, and 1000 nM) for 24 h, and the % of apoptotic cells was significantly and dose-dependently increased. Data are reported as means ± SD (by one-way ANOVA). * *p* < 0.05 vs. Control.

In the first pATC culture with STRN–ALK fusion, crizotinib induced a significant reduction (*p* < 0.05, ANOVA; vs. Control) in viability/proliferation (Figure 1A), in accordance with the cell counting data (see Table 1).

**Figure 2:**
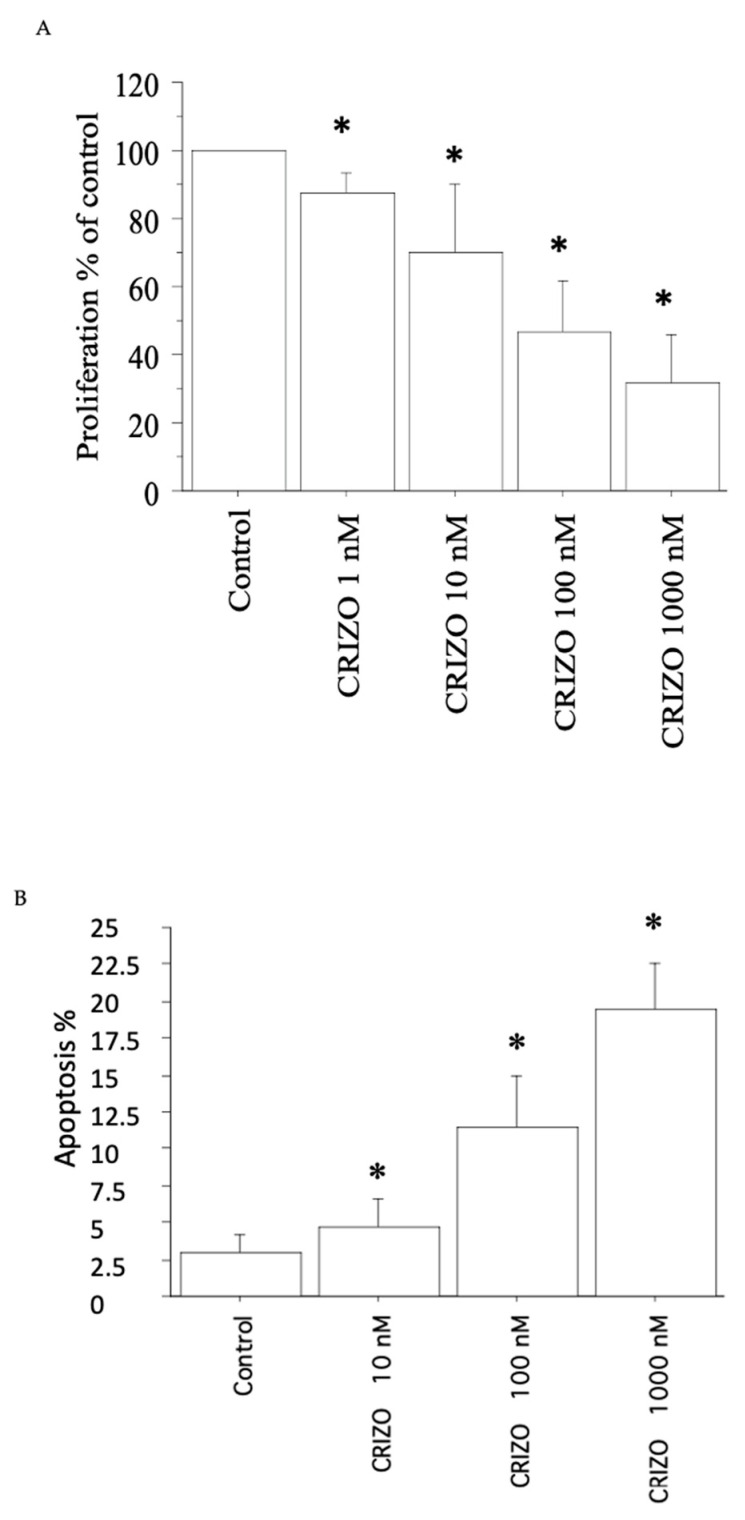
Cell proliferation in the second pATC culture with STRN–ALK fusion. (**A**) WST-1 assay in pATCs treated for 24 h with crizotinib (1, 10, 100, and 1000 nM). Proliferation was significantly reduced vs. Control. Bars represent means ± SD. * *p* < 0.05 vs. Control. (**B**) Apoptosis was shown by Hoechst 33342 staining in pATCs after treatment with crizotinib (10, 100, and 1000 nM) for 24 h, and the % of apoptotic cells was significantly and dose-dependently increased. Data are reported as means ± SD (by one-way ANOVA). * *p* < 0.05 vs. Control.

In the second pATC culture with STRN–ALK fusion, crizotinib significantly (*p* < 0.05, ANOVA) induced a reduction (vs. Control) in viability/proliferation (Figure 2A), in accordance with the cell counting data (see Table 1).

**Figure 3:**
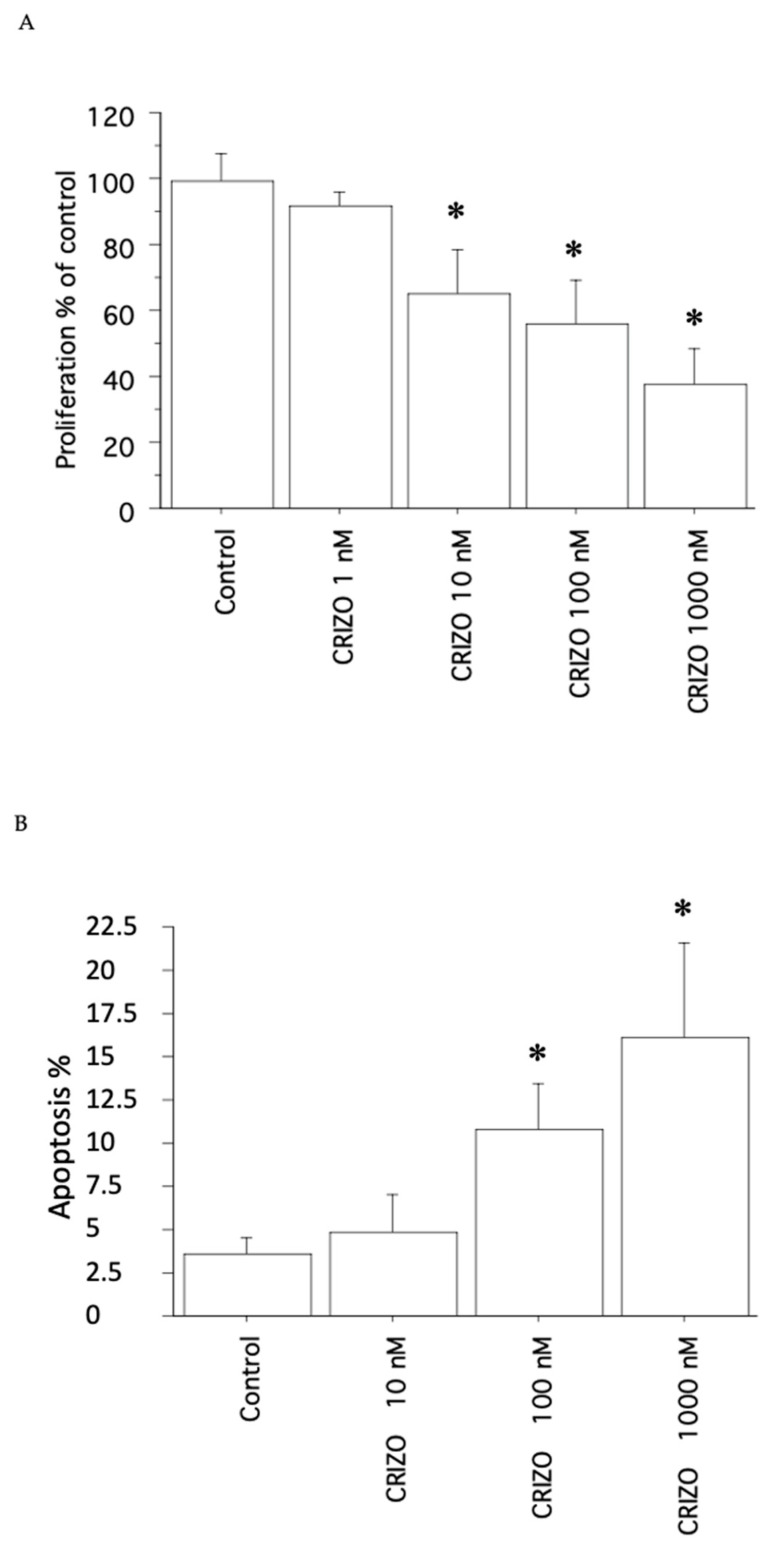
Cell proliferation in pATC culture without STRN–ALK fusion. (**A**) WST-1 assay in pATCs treated for 24 h with crizotinib (1, 10, 100, and 1000 nM). Proliferation was significantly decreased vs. Control. Bars represent means ± SD. * *p* < 0.05 vs. Control. (**B**) Apoptosis was shown by Hoechst 33342 staining in pATCs after treatment with crizotinib (10, 100, and 1000 nM) for 24 h, and the % of apoptotic cells was significantly and dose-dependently increased. Data are reported as means ± SD (by one-way ANOVA). * *p* < 0.05 vs. Control.

In the pATC culture without STRN–ALK fusion, crizotinib induced a significant reduction (*p* < 0.05, ANOVA) (vs. Control) in viability/proliferation (Figure 3A), in agreement with the cell counting data (see Table 1); however, the effect was slightly lower than that observed in the presence of the STRN–ALK fusion.

Moreover, in normal TFCs, crizotinib showed a slight but significant reduction in proliferation vs. Control (*p* < 0.05, ANOVA), with Crizotinib 100 and 1000 nM. The cell counting data agreed with the above-mentioned data.

**Figure 4:**
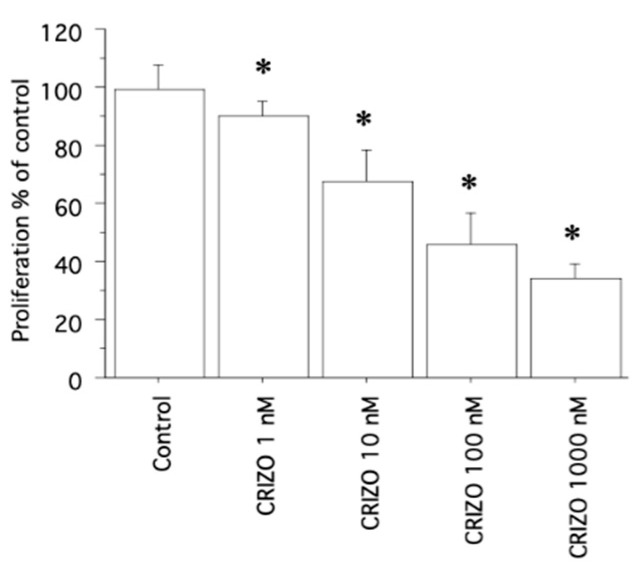
Cell proliferation in AF cells treated with crizotinib (1, 10, 100, and 1000 nM) for 24 h, by WST-1 assay. Proliferation was significantly reduced vs. Control. Bars represent means ± SD. * *p* < 0.05 vs. Control.

In AF cells, crizotinib significantly decreased viability/proliferation (vs. Control; *p* < 0.05, ANOVA) (Figure 4), which is also in accordance with cell counting data.

### 2.2. Apoptosis

Upon treating the first pATC culture with STRN–ALK fusion with crizotinib (10, 100, and 1000 nM), apoptotic cells (expressed as a %) increased dose-dependently. Upon treating cells with Crizotinib 10 nM, 3% of the cells were apoptotic; the apoptotic rate increased to 10% and 18%, respectively, with Crizotinib 100 and 1000 nM (*p* < 0.05, ANOVA; Figure 1B). Annexin V staining supported these results.

Upon treating the second pATC culture with STRN–ALK fusion with crizotinib (10, 100, and 1000 nM), apoptotic cells increased dose-dependently. Crizotinib 10, 100, and 1000 nM increased the apoptotic rate vs. Control to 4%, 11%, and 18%, respectively (*p* < 0.05, ANOVA; Figure 2B). Annexin V staining confirmed these results.

In the pATC culture without STRN–ALK fusion, the treatment with crizotinib (10, 100, and 1000 nM) increased the apoptotic rate vs. Control, even if slightly in comparison to the results obtained in the presence of STRN–ALK fusion (*p* < 0.05, ANOVA; Figure 3B). Annexin V staining reinforced these results.

### 2.3. Migration and Invasion Assays

**Figure 5:**
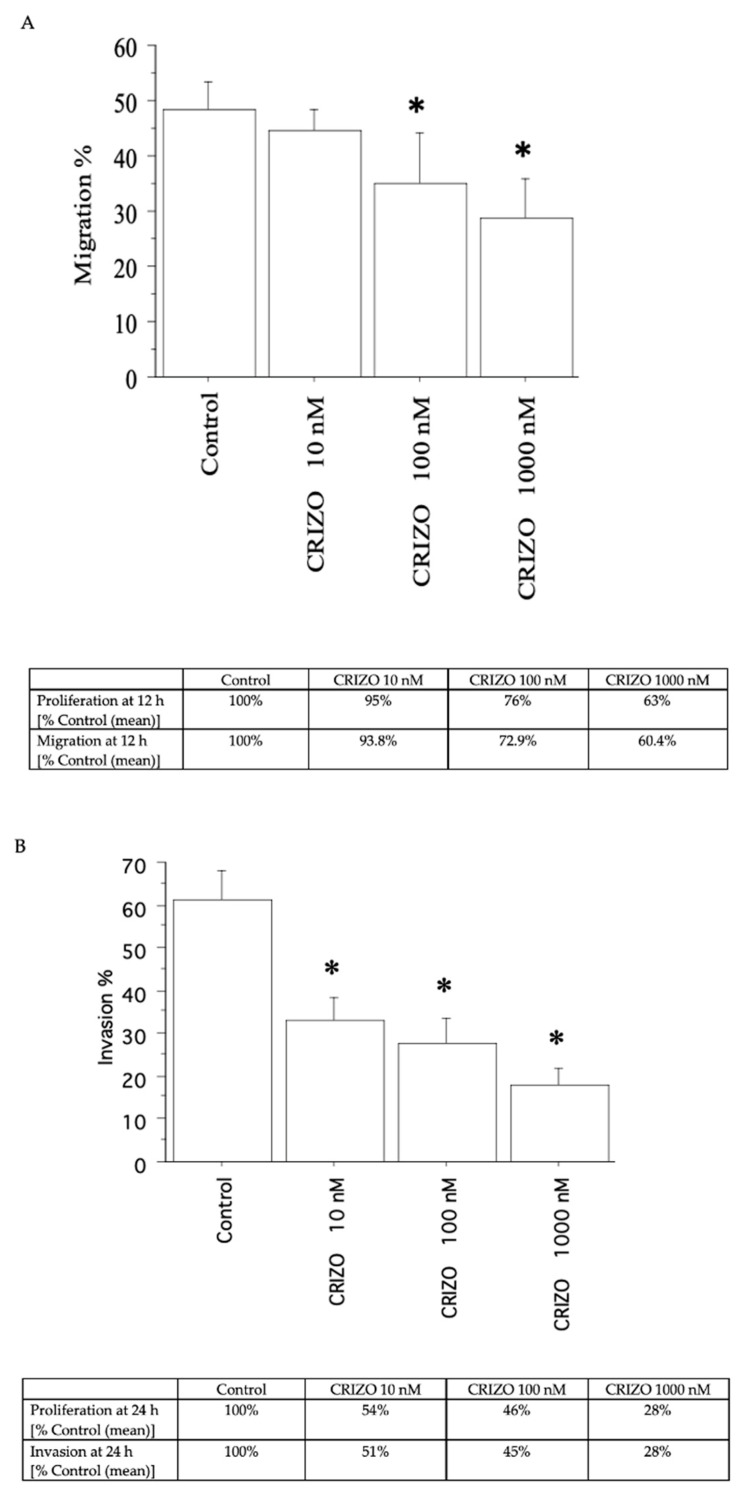
Migration and invasion in the first pATC culture with STRN–ALK fusion. The migration test was carried out for 12 h (**A**) and invasion for 24 h. (**B**) For comparison, the inhibition of proliferation (as a % of Control) at 12 h for migration and at 24 h for invasion is reported in the tables below each panel. Bars are means ± SD. * *p* < 0.05 vs. Control (represented by medium + FCS 10%) by Newman–Keuls test.

**Figure 6:**
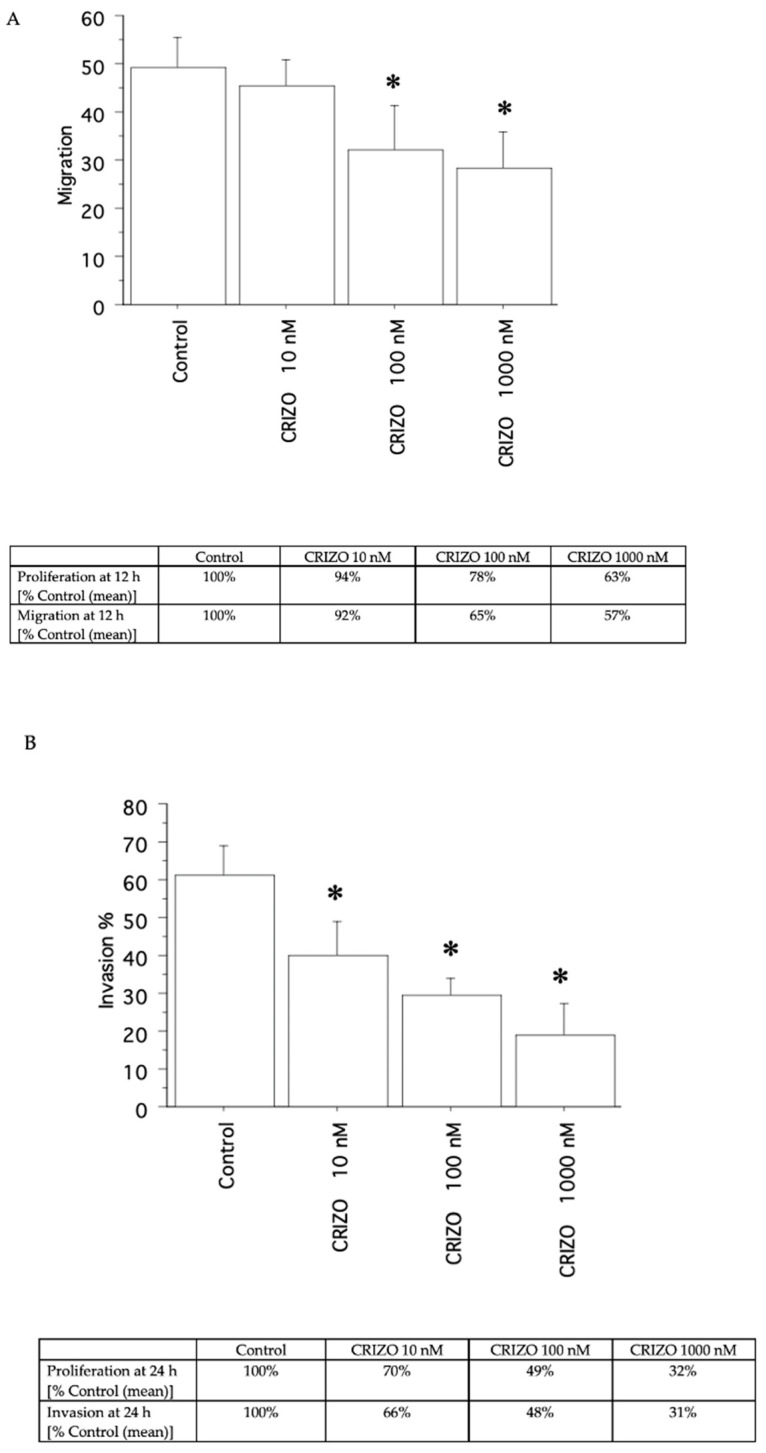
Migration and invasion in the second pATC culture with STRN–ALK fusion. The migration test was carried out for 12 h (**A**) and invasion for 24 h. (**B**) For comparison, the inhibition of proliferation (as a % of Control) at 12 h for migration and at 24 h for invasion is reported in the tables below each panel. Bars are means ± SD. * *p* < 0.05 vs. Control (represented by medium + FCS 10%) by Newman–Keuls test.

**Figure 7:**
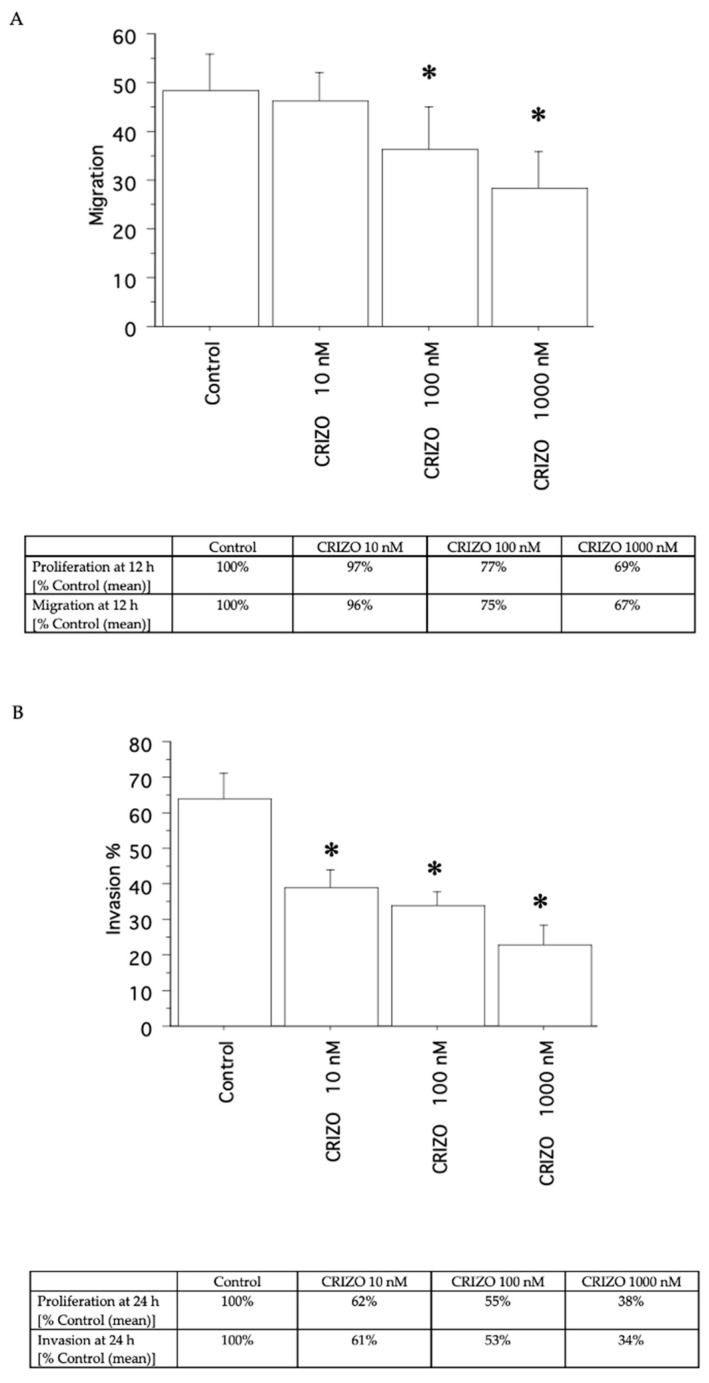
Migration and invasion in the pATC culture without STRN–ALK fusion. The migration test was carried out for 12 h (**A**) and invasion for 24 h. (**B**) For comparison, the inhibition of proliferation (as a % of Control) at 12 h for migration and at 24 h for invasion is reported in the tables below each panel. Bars are means ± SD. * *p* < 0.05 vs. Control (represented by medium + FCS 10%) by Newman–Keuls test.

A decrease in migration (Figure 5A and Figure 6A) and invasion (Figure 5B and Figure 6B) was shown by crizotinib (10, 100, and 1000 nM), in the two pATC cultures with STRN–ALK fusion and in that without STRN–ALK fusion, but only slightly (Figure 7A,B).

## 3. Discussion

ATC is one of the deadliest human cancers and its conventional treatment includes surgical debulking, accelerated hyperfractionated external beam radiation therapy, and chemotherapy, mainly with doxorubicin or cisplatin, which permit to achieve ~10 months of median survival. Therefore, it could be useful to determine an effective systemic treatment to ameliorate the quality of life of these patients [20]. The new knowledge of the molecular pathways involved in the development of ATC has given rise to the realization of novel drugs.

In the management of thyroid nodules and cancers, ALK fusions represent both a diagnostic marker and a possible therapeutic target [39].

Crizotinib is an oral small-molecule TKI of the ALK, ROS1, and MET kinases, approved in ALK-positive NSCLC. The role of ALK as a therapeutic target has been evaluated by different case reports of patients with ALK-positive TC, including cases of metastatic and RAI-resistant cancer treated with crizotinib [45,46,68]. Moreover, in locally advanced NSCLC, neoadjuvant crizotinib may be feasible and well tolerated for complete resection. Crizotinib therapy before surgery may provide thorough elimination of circulating molecular residual disease and not influence the reuse of first-line crizotinib [69]. On the other hand, little is known about neoadjuvant treatment in ALK-positive ATC. Recently, it was shown that the neoadjuvant use of dabrafenib and trametinib followed by surgery achieved 24-month OS rates of 80% [70].

A case of an ATC patient with ALK with a good response to crizotinib treatment is represented by a 71-year-old woman with no history of exposure to ionizing radiation, with a rapidly growing right cervical tumor and mild dysphagia [45]. The patient had an ALK rearrangement in both the well-differentiated and the anaplastic sections of the tumor, and the presence of the ALK rearrangement in these components probably led it to be an early carcinogenic driver event [45].

A multicenter, open-label, single-arm phase 1b study (PROFILE 1013; NCT01121588), which has now terminated, was conducted in patients with ALK-positive advanced malignancies other than NSCLC [71]. Patients received an initial dose of crizotinib 250 mg twice daily. Primary endpoints were safety and objective responses according to National Cancer Institute International Response Criteria or Response Evaluation Criteria in Solid Tumors version 1.1. A total of 44 patients participated in the study (lymphoma, n = 18; inflammatory myofibroblastic tumors (IMTs), n = 9; other tumors, n = 17). The objective response rate was 53% for lymphoma, with one partial response (PR) and eight complete responses (CRs); 67% for IMTs, with one CR and five PRs; and 12% for other tumors, with two PRs for colon carcinoma and MTC, respectively. The median duration of the therapy was about 3 years for patients with IMTs and lymphoma, with progression-free survival at 2 years of 67% and 63%, respectively [71,72].

Moreover, more recently, a preclinical study [73] showed that the ALK fusion downregulated NIS and that this could lead in thyrocytes to direct inhibition of ALK or its downstream signaling pathways. Nis downregulation occurred early in ALK-driven thyroid carcinogenesis, as well as in well-differentiated TC, and it is completely lost in PDTC. Acute STRN–ALK expression in thyrocytes leads to increased JAK/STAT3, PI3K/AKT/mTOR, and MAPK signaling outputs linked to the downregulation of most of thyroid differentiation and iodine metabolism/transport genes, such as Foxe1, Slc5a5 (Nis), Slc5a8, Dio1, Duox1/2, Duoxa2, Tg, and Glis3. Furthermore, the STRN–ALK-induced downregulation of NIS caused a strong reduction in RAI uptake, which was reversed by the ALK inhibitors crizotinib and ceritinib [73].

Our study demonstrates for the first time the antineoplastic activity of crizotinib in primary human ATC cells (mainly with STRN–ALK) obtained from thyroid ATC tissue samples, showing that it inhibits pATC proliferation in vitro, as well as the migration and invasion capabilities of these cells, and it increases apoptosis.

Since, so far, the usual treatments in ATC patients permit to reach about 6–10 months of median survival and it is challenging to predict ATC patient clinical therapy responsiveness, it could be useful to identify a valid systemic treatment to improve the quality of life of these patients. The possibility to evaluate in each patient the molecular pathways involved in the development of ATC and to test in vitro different TKIs can help to develop new personalized therapies and not to use ineffective drugs [74]. In this regard, primary human cell cultures have some convenience with respect to continuous cells for the evaluation of the antineoplastic effect of drugs. Thyroid continuous cell lines have been commonly used as preclinical models for research aims [75], but primary cells have phenotypic features that resemble the original tumor; on the other hand, immortalized cell lines, even if they are easy to handle, generally adapt to the in vitro growth conditions and lose their primitive intrinsic characteristics [74]. Therefore, primary cells can be used in vitro to investigate cell chemosensitivity in each subject and can give indications to clinicians regarding which could be the right therapy, to avoid inactive compounds.

Personalized medicine, which is focused on both patients and their disease features, could be considered the future of the treatment approach, mainly in diseases such as anaplastic thyroid cancer, which represent a challenge from a therapeutic point of view.

## 4. Materials and Methods

### 4.1. Cell Cultures

#### 4.1.1. Tissue Samples

Thyroid tissue samples were gathered from twelve ATC patients (age range, 54–82; seven females, five males; tumor size range, 5–15 cm) at surgery and from six healthy controls (who had undergone parathyroidectomy), in order to compare primary cell cultures obtained from malignant cancer tissues and those from normal tissues. The diagnosis of ATC was made in accordance with standard histological, laboratory, and clinical criteria [76,77,78].

All subjects agreed to be involved in the study, which was approved by the Ethics Committee of University of Pisa.

#### 4.1.2. Characterization of ATC Samples

The expression of TSH receptor, thyroperoxidase (TPO), thyroglobulin (Tg), and sodium/iodide symporter (NIS) was determined by immunohistochemistry.

Direct DNA sequencing, microdissection and DNA extraction, and determination of BRAF mutation were performed in accordance with standard methods previously reported [76,77,78]. Mutations in BRAF, N-RAS, H-RAS, K-RAS, RET/PTC1, RET/PTC3, and PAX8/PPARγ rearrangements were detected through real-time RT-PCR [79,80]. STRN–ALK fusion was detected as previously reported [39].

#### 4.1.3. Primary Cell Cultures

Tumor tissues were thinly cut and then washed in M-199 medium containing 500,000 U/L penicillin, 1,000,000 U/L nystatin, and 500,000 U/L streptomycin. Then, samples were put into DMEM medium with fetal calf serum (FCS; 10%) and kept at 37 °C in 5% CO_2_. After confluence was reached, cells were detached and seeded in Methocel at the 3rd passage to investigate the colony-forming efficiency; then, they were expanded in flasks [76,77,78].

Normal thyroid follicular cell cultures (TFCs) were established as described previously [79], in order to compare primary cell cultures obtained from malignant cancer tissues and those from normal tissues.

Cells were tested for chemosensitivity at the 4th passage [61,63].

#### 4.1.4. AF Cell Line

As positive control, the AF cell line is a continuous cell line obtained from primary ATC cells that could be passed over 50 times. AF cells grew in nu/nu mice when inoculated sc [63].

### 4.2. Proliferation, Cell Viability, and Cytotoxicity Assay

Cell Proliferation Reagent WST-1 (Sigma-Aldrich, Merck, Darmstadt, Germany) was performed to evaluate proliferation, cell viability, and cytotoxicity [76,77,79]. An increase in the number of viable cells leads to the increase in the activity of cellular mitochondrial dehydrogenases, which cleave tetrazolium salts to formazan, which was quantified by measuring the absorbance of the dye solution at 450 nm through an ELISA reader.

Cells were seeded (35,000 cells/mL in 100 μL per well) in a 96-well microtiter plate, and crizotinib (1, 10, 100, and 1000 nM) or dimethyl sulfoxide (the vehicle, as negative control) alone were added in quadruplicate to the wells. After 24 h, 10 μL of WST-1 Reagent was put in the wells, and the absorbance was read at 450 nm vs. Control (the same cells with no treatments). As the blank (culture medium plus WST-1, with no cells), 100 μL of culture medium and 10 μL of WST-1 were added to one well. The determination of the concentration of crizotinib necessary to obtain 50% inhibition of growth (IC50) was evaluated by linear interpolation.

The absorbance of control and treatments was normalized with respect to the blank. The Control was expressed as 100% and treatments as a percentage of it.

### 4.3. Cell Counting

Since the proliferation, cell viability, and cytotoxicity assay measures mitochondrial cell activity and since a direct correlation with the cell number does not always exist, cell number counting through a hemocytometer was also used to detect proliferation [76,77,79]. The IC50 was obtained by linear regression analysis of the dose–response curve.

### 4.4. Apoptosis

#### 4.4.1. Hoechst 33342 Uptake

pATCs were seeded (in 100 μL per well, 35,000 cells/mL), treated for 24 h (at 37 °C, 5% CO_2_) with crizotinib (1, 10, 100, and 1000 nM), and then stained with Hoechst [79]. The ratio of apoptotic/total cells × 100 was calculated (apoptosis index) for treatments and Control (the same cells with no treatments).

#### 4.4.2. Annexin V

pATCs were plated in Lab-TekII Chamber Slide (Nalge Nunc International, ThermoFisher Scientific, Waltham, MA, USA), then treated for 24 h with crizotinib, and evaluated by Annexin V binding assay [79].

### 4.5. Migration and Invasion Tests

These tests were performed in 96-well Transwell Permeable Supports (Corning Life Sciences, Sigma-Aldrich, Merck, Darmstadt, Germany) in accordance with the manufacturer’s suggestions [63,81]. Cells were starved for 5 h in serum-free medium through a PBS solution with 5 mM EDTA. Cells were counted, centrifuged, and then placed on a plate (0.5 × 10^5^ cells/well) in medium in the absence of serum.

To create a gradient, 10% *v*/*v* FCS (or serum-free medium as negative control) was added to receiver wells with increasing concentrations of crizotinib; then, the media were removed from the lower compartments, and calcein AM (2 µg/mL; Sigma-Aldrich) was added for 1 h. The intracellular fluorescence was evaluated by ELISA (at 485 nm for excitation and 520 nm for emission).

Migration was carried out for 12 h and invasion for 24 h as previously reported [63,81]. A standard curve was performed to turn fluorescence readings into the number of migrated/invasive cells.

### 4.6. Statistical Analysis

The experiments were performed three times with the primary cells obtained from each subject (the mean of the different samples is shown). The results are reported as means ± standard deviation (SD) or as medians and interquartile range for normally distributed variables. One-way ANOVA, or the Mann–Whitney U or Kruskal–Wallis test, was performed to relate the mean group values for normally distributed variables, and to compare proportions, we used the χ2 test. The Bonferroni–Dunn test was applied to post hoc comparisons on normally distributed variables. One-way ANOVA and Newman–Keuls multiple comparisons test were used for apoptosis.

## 5. Conclusions

ATC is one of the deadliest human cancers, with a low overall survival rate and a mean survival of ~3.2 years and 6 months, respectively [15]. Until now, the usual treatments in ATC patients permit to reach about 6–10 months of median survival. For these reasons, it is challenging to predict ATC patient clinical therapy responsiveness, and it could be useful to identify a valid systemic treatment to improve the quality of life of these patients.

ALK fusions are considered both a diagnostic marker and a possible therapeutic target in ATC, and our in vitro study demonstrates for the first time the antineoplastic action of crizotinib in primary human ATC cells (mainly with STRN–ALK) obtained from thyroid ATC tissue samples, reporting in vitro that it inhibits pATC growth, as well as the migration and invasion capabilities of these cells, and it increases apoptosis.

In conclusion, the antineoplastic activity of crizotinib has been shown in human pATCs (with STRN–ALK) in preclinical studies in vitro; further studies are necessary to confirm these data, opening the way to future clinical evaluation in these patients.

The expanding knowledge of the molecular biology in ATC and the ongoing clinical trials give hope for the development of further therapeutic options.

The chance to test in each patient the sensitivity to multiple TKIs in vitro could build on progress leading to the new era of personalized treatments, avoiding the administration of ineffective compounds and ameliorating the quality of life of these patients.

## Figures and Tables

**Table 1:** Cell number per well (and % with respect to Control) in the three primary human ATC cell (pATC) cultures with/without STRN–ALK fusion, according to the treatments.

	Control	Crizotinib 1 nM	Crizotinib 10 nM	Crizotinib 100 nM	Crizotinib 1000 nM	*p* (* by ANOVA)	IC50
First pATC culture with STRN–ALK fusion	19,994 ± 9870 (100%)	17,195 ± 998 (86%)	10,997 ± 943 (55%)	9797 ± 1005 (49%)	4998 ± 1018 (25%)	<0.01	72 nM
Second pATC culture with STRN–ALK fusion	20,005 ± 9745 (100%)	18,005 ± 1102 (90%)	13,605 ± 988 (68%)	9202 ± 878 (46%)	6602 ± 988 (33%)	<0.01	85 nM
pATC culture without STRN–ALK fusion	19,898 ± 9470 (100%)	18,903 ± 1114 (95%)	12,734 ± 990 (64%)	11,540 ± 998 (58%)	7959 ± 974 (40%)	<0.01	112 nM

* *p* < 0.01 of Crizotinib 1000 nM vs. Control.

## Data Availability

The data presented in this study are available within the article.
